# Extramedullary Imaging Manifestations of Leukemia/Lymphoblastic Lymphoma in Pediatrics: A Case Series

**DOI:** 10.7759/cureus.54714

**Published:** 2024-02-22

**Authors:** Marcia Mejia, Mónica Royero Arias, Vanessa Santiago Pacheco, Jonathan Pimiento Figueroa

**Affiliations:** 1 Radiology, Universidad de Antioquia, Medellín, COL; 2 Pediatric Radiology, Servicios de Salud San Vicente Fundación, Medellín, COL; 3 Pathology, Universidad de Antioquia, Medellín, COL; 4 Radiology, Servicios de Salud San Vicente Fundación, Medellín, COL

**Keywords:** hematologic neoplasms, pediatrics, diagnostic imaging, precursor b-cell lymphoblastic leukemia, leukemia

## Abstract

Leukemia is the most common childhood malignancy and often presents with nonspecific symptoms. Occasionally, it presents with extramedullary manifestations, which have been more frequent in cases of myeloid lineage or T cells. However, precursor B-cell leukemia/lymphoblastic lymphoma can also have extramedullary manifestations in some patients. Describing certain clinical features along with diagnostic imaging can establish a presentation pattern and suggest a diagnosis in the pediatric population. Herein, we present a series of four patients with extramedullary manifestations of B-cell lymphoblastic leukemia, describing their clinical imaging and histopathological characteristics.

## Introduction

Leukemia is the most common neoplasm in the pediatric population, accounting for approximately one-third of childhood cancers, 80-85% of which originate from B cells and the rest from T cells [1]. B-cell derivatives manifest as pure acute lymphoblastic leukemia (ALL-B) in 80% of cases while the remaining 20% may present with extramedullary disease or lymphoblastic lymphoma (LLB-B) [2]. Lymphoblastic lymphoma can occur in half of the patients either in isolation or in a mixed pattern with leukemia (ALL-B/LLB) in the other half [1,2]. According to the fifth edition of the World Health Organization's (WHO's) classification for hematologic tumors, B-cell leukemia and lymphoblastic lymphoma correspond to parts of the same disease spectrum, with a cutoff of more than 25% lymphoblasts in the bone marrow defining leukemia and less than 25% defining lymphoblastic lymphoma [3]. Symptoms vary, especially in cases with extramedullary involvement, which can lead to delays in diagnosis and treatment [4]. While the definitive confirmation of this disease is established through immunophenotypic and histopathological studies, imaging findings can suggest a diagnosis and can be used for monitoring. Here, we present a series of four cases, emphasizing the imaging findings.

## Case presentation

Case 1

**Figure 1:**
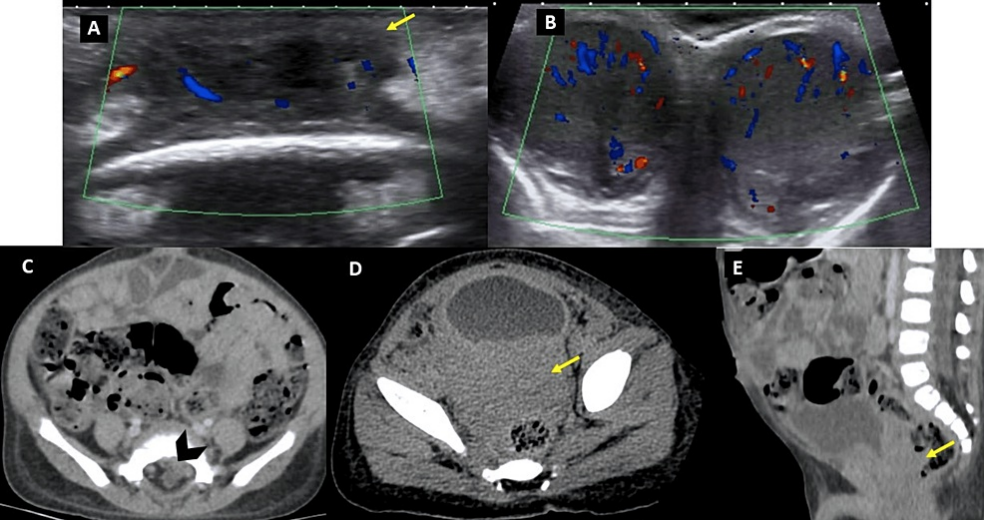
(A) Soft tissue ultrasound of the right frontal region showing a solid, hypoechoic mass with increased color Doppler flow, located in the subcutaneous tissue (yellow arrow). (B) Testicular ultrasound showing increased volume and color Doppler flow in both testicles, with hypoechogenicity of the parenchyma. (C) Axial abdomen and pelvis CT scan showing a solid mass in the left extradural intra-spinal space at the S1 level (yellow arrow). (D,E) Axial and sagittal abdomen and pelvis CT scan showing a solid mass without contrast enhancement located retrovesically with vesicoprostatic infiltration (yellow arrows).

A 22-month-old male patient presented with a clinical history of four months characterized by the appearance of multiple violaceous masses in the soft tissues of the frontal region, scalp, retroauricular, and perianal regions, associated with pallor and an increase in the size of both testicles. The initial clinical suspicion was multiple hemangiomas. Upon admission, laboratory tests revealed pancytopenia. Imaging studies showed bilateral testicular enlargement and increased flow on color Doppler evaluation, multiple masses in the soft tissues of the head (the largest in the frontal region), a mass in the retrovesical space, and an intraspinal epidural lesion (Figure 1).

**Figure 2:**
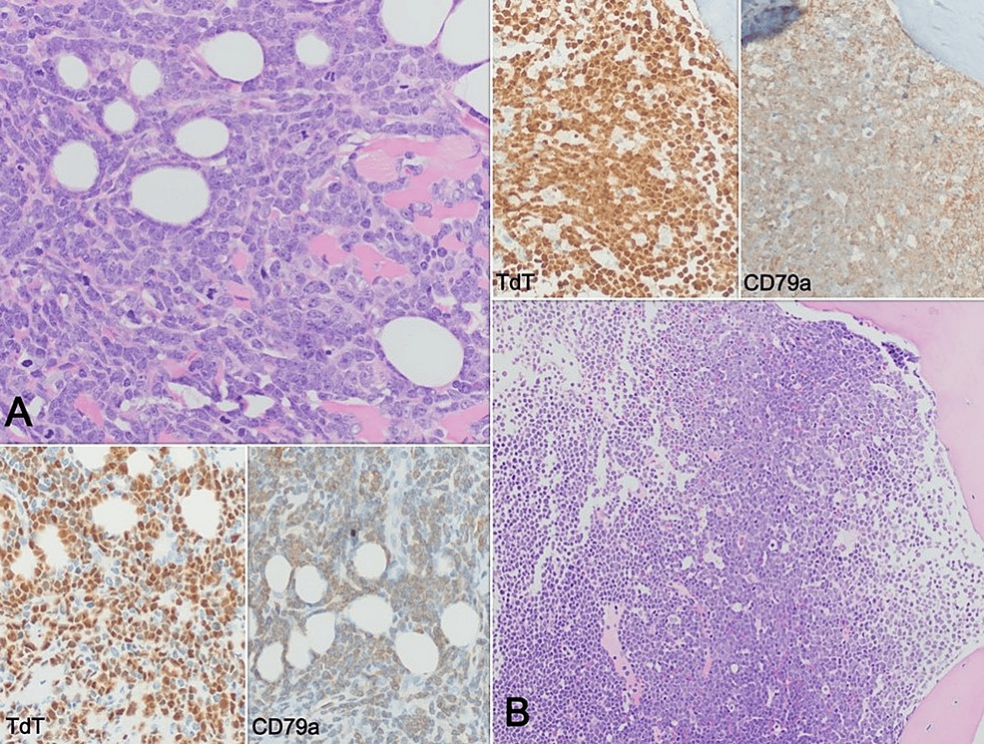
(A) H-E 40x Frontal mass biopsy showing extensive involvement of soft tissues by a neoplasm of immature lymphocytes, with scant cytoplasm and open chromatin, abundant associated mitoses, invading adipose tissue and skeletal muscle. (B) H-E 20x Bone marrow biopsy showing involvement by blasts with similar characteristics. Immunohistochemistry for TdT (40x) and CD79a (40x) in both samples confirms the immature B lineage of the neoplastic cells.

Based on the imaging findings and laboratory tests, a suspicion of lymphoproliferative disease vs. neuroblastoma was raised. Bone marrow aspiration and biopsy revealed the presence of 85% lymphoblasts with an immunophenotype by flow cytometry showing: cyCD79a+, CD19+, CD38+, heterogeneous CD20, CD10-, and CD34-. These findings were confirmed by immunohistochemistry. Additionally, a biopsy of the frontal mass confirmed involvement by a neoplasm with similar morphological and immunophenotypic characteristics (Figure 2).

Further studies demonstrated a 46,XY karyotype with t(4;11), KMT2A gene rearrangement. This led to the diagnosis of B-cell lymphoblastic leukemia/lymphoma with KMT2A rearrangement.

Case 2

**Figure 3:**
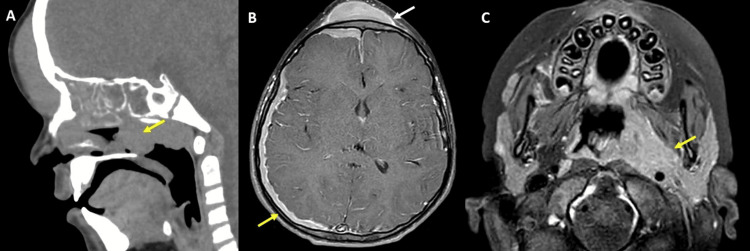
(A) Sagittal plain neck CT scan showing a solid frontal mass and thickening of the nasopharyngeal soft tissues (yellow arrow). (B) Contrast-enhanced axial brain MRI showing a solid mass with homogeneous enhancement located in the frontal epicranial soft tissue (white arrow), associated with pachymeningenal thickening and enhancement of the right cerebral hemisphere (yellow arrow). (C) Solid, multi-space-occupying mass showing homogeneous enhancement involving the nasopharynx, parapharyngeal space, carotid, and masticatory space on the left side (yellow arrow).

**Figure 4:**
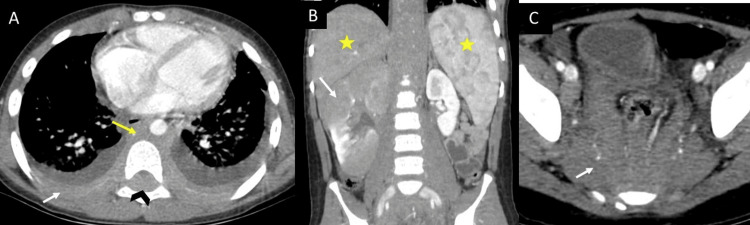
(A) Contrast-enhanced chest CT scan in axial section showing infiltrative involvement of the paravertebral mediastinum (yellow arrow) extending into both pleural cavities with pleural effusion and thickening (white arrow), and extending into the epidural space of the spinal canal (black arrowhead). Contrast-enhanced abdomen and pelvis CT scan in coronal (B) and axial (C) sections showing hepatosplenomegaly (yellow stars in B), right nephromegaly with multiple ill-defined hypodense lesions in the kidney and perirenal space (white arrow in B). Infiltrative-looking mass in the retroperitoneum, presacral space, and pelvic side walls (white arrow in C).

A six-year-old male patient presented with a clinical picture of frontal region trauma. He was discharged but returned 20 days later due to an increase in the size of the swelling in the frontal region, accompanied by asthenia and abdominal pain. Upon admission, laboratory tests revealed pancytopenia and elevated C-reactive protein (CRP). A plain head CT scan showed a solid mass (42 HU) in the frontal soft tissues and another mass with similar behavior and multi-compartmental in the neck. Therefore, additional studies, including contrast-enhanced brain MRI and contrast-enhanced thoracoabdominal CT, were performed. Additional studies revealed pachymeningeal thickening and enhancement, bilateral pleural thickening and effusion, abdominal organomegaly, and multiple masses (Figures 3, 4).

Bone marrow aspiration and biopsy were performed. Flow cytometry immunophenotyping demonstrated 90% B lymphoblasts, intermediate CD45+, strong CD34+, heterogeneous CD19+, CD79a+, TdT+, CD10+, CD66c-/+, CD81+, and negative CD20. These findings were confirmed by immunohistochemistry. Furthermore, involvement of B lymphoblastic leukemia in the central nervous system was demonstrated in the cerebrospinal fluid sample. Cytogenetic studies revealed a 46,XY karyotype, del (9)(p13p21)34/47. This led to the diagnosis of B lymphoblastic lymphoma/leukemia, NOS.

Case 3

**Figure 5:**
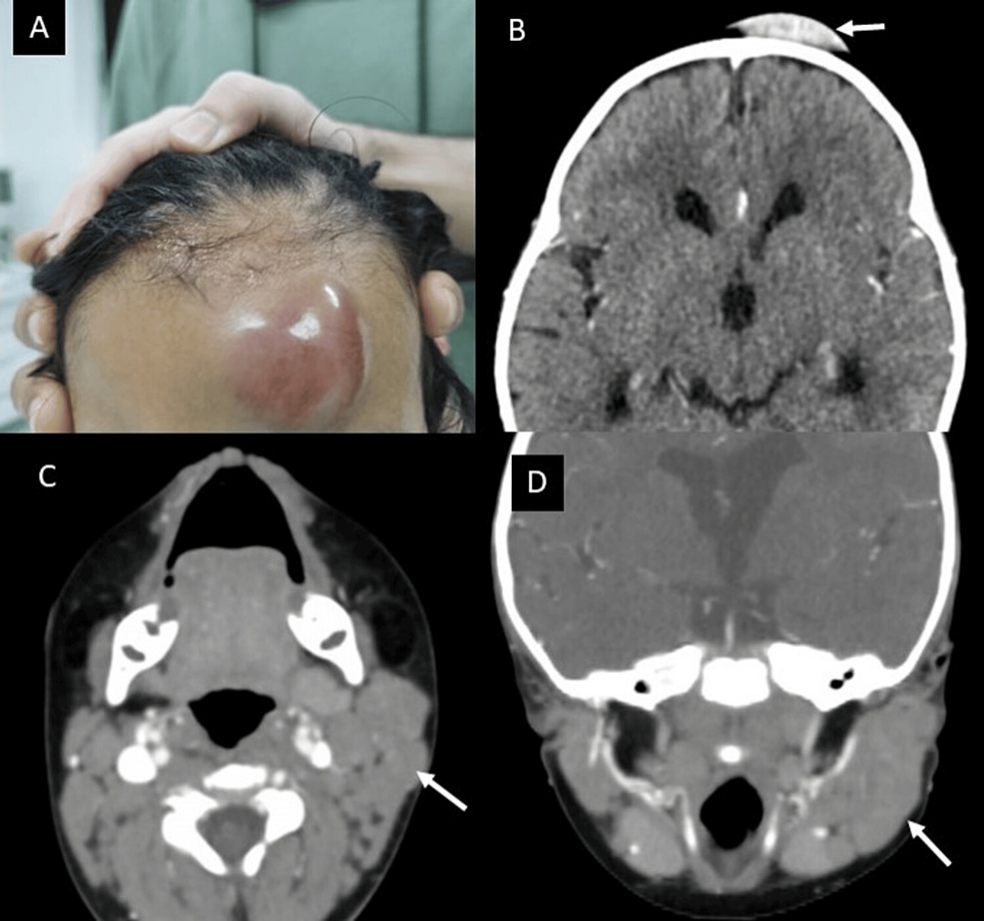
(A) Photograph of a violet frontal mass on the left side. (B) Contrast-enhanced axial head CT scan showing a mass in the left frontal epicranial soft tissues with homogeneous enhancement after contrast administration (white arrow). (C) Contrast-enhanced neck CT scan in axial section showing lymphadenopathies in stations IIa and IIb on the left side (white arrow). (D) Contrast-enhanced neck CT scan in coronal section showing left parotidomegaly (white arrow).

A 22-month female patient presented with a clinical picture of a one-month history of a mass in the frontal region, followed by the development of other masses in the submandibular and preauricular regions. The initial clinical suspicion was skull osteomyelitis. Upon admission, laboratory tests showed neutropenia, thrombocytopenia, and an increase in immature cells in peripheral blood: neutrophils 100 cells/mcL, platelets 70.000 cells/mcL, and 20% of blasts in peripheral blood. Imaging studies revealed a mass in the soft tissues of the left frontal region, parotidomegaly, and multiple cervical lymphadenopathies (Figure 5).

Bone marrow aspiration and biopsy demonstrated the presence of 75% B lymphoblasts, CD19+, CD20-, heterogeneous CD10, CD34-, CD38+, CD66c/CD123-, CD81-, and weak CD45, CD3-, CD79a+. These findings were confirmed by immunohistochemistry. Conventional cytogenetics showed a normal karyotype of 46,XX, with fluorescence in situ hybridization (FISH) indicating an extra signal for KMT2A. This confirmed the diagnosis of B lymphoblastic lymphoma/leukemia with KMT2A rearrangement, without central nervous system involvement in the cerebrospinal fluid study.

Case 4

**Figure 6:**
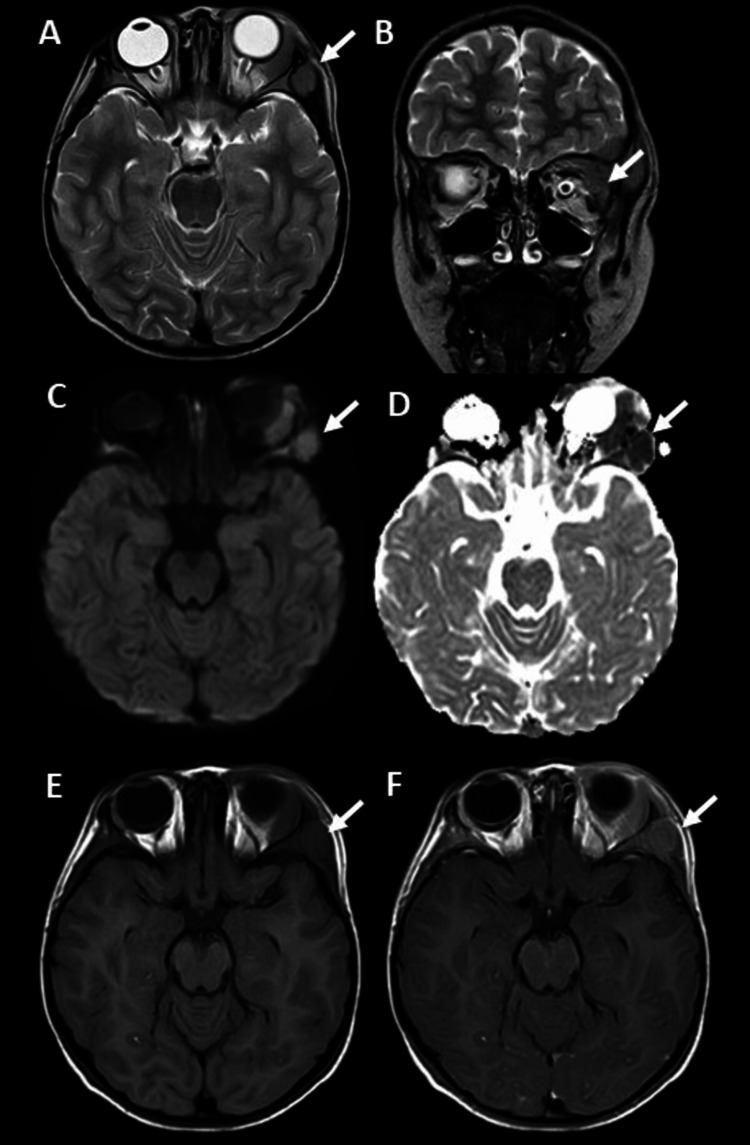
Contrast-enhanced brain MRI in T2-weighted axial and coronal sequences (A and B) showing a hypointense mass in the left frontal bone extending into the extra-axial frontal space, intra and extraconal orbit, and periorbital soft tissues (white arrows). The mass restricts in diffusion sequences, appearing hyperintense in b1000 (white arrow in C) and hypointense in ADC (white arrow in D). It is hypointense in the pre-contrast T1 sequence (white arrow in E), with post-contrast enhancement (white arrow in F). ADC: apparent diffusion coefficient

A five-year-old male patient presented with a one-month history of edema and pain in the left periorbital and supraorbital region. Upon admission, laboratory tests showed anemia, thrombocytopenia, and neutropenia: hemoglobin 5.3 g/dl, hematocrit 16.5%, platelets 82000 cells/mcL, and neutrophils 910 cells/mcL. Contrast-enhanced brain MRI imaging revealed a mass in the left frontal bone, extending into the orbit and periorbital soft tissues (Figure 6).

Based on contrast-enhanced brain MRI imaging and laboratory findings, a lymphoproliferative disease was suspected. Bone marrow aspiration and biopsy identified 63% B lymphoblasts and intermediate CD45, CD19+, CD10+, CD34+, CD38+, TdT+, CD20-, CD66c-. These findings were confirmed by immunohistochemistry. No evidence of BCR: ABL1 fusion or KMT2A rearrangements was found in cytogenetics or FISH. This led to the diagnosis of B lymphoblastic lymphoma/leukemia, without further classification. No central nervous system involvement was observed in the cerebrospinal fluid study.

## Discussion

B-cell acute lymphoblastic leukemia/lymphoma is a neoplasm of hematopoietic precursors with B-lineage differentiation [1]. It most commonly occurs in patients under six years of age and in males (with a male-to-female ratio of 1.9:1) [2]. The five-year survival rate is 90% in pediatric patients, with a relapse rate of approximately 15-20% [4,5]. According to consensus, the term "leukemia" is used when the main involvement is in peripheral blood and bone marrow, and the term "lymphoma" is used if the primary site of involvement is in lymph nodes or extranodal sites, with an arbitrary distinction if there is both medullary and extramedullary involvement [2,3].

Morphological and histopathological studies are essential but not sufficient for the initial approach. A complete diagnosis can be made from a peripheral blood sample in the presence of circulating blasts. Bone marrow examination is essential in cases without circulating blasts and during follow-up. Morphological findings are similar in most cases, demonstrating blasts with scant cytoplasm and immature chromatin; these blasts infiltrate the affected tissue and alter its architecture, both in the bone marrow and in the affected extramedullary tissue. The essential immunophenotype for diagnosis is specific to B-lineage, with positivity for CD19, CD79a, expression of an immature marker, such as TdT and CD34, and variable expression of CD10, CD38, CD20, CD66c, CD81, CD9, and CD123. Flow cytometry immunophenotyping provides a rapid initial diagnosis that allows treatment protocols to be initiated while a final diagnosis is made based on cytogenetic and molecular biology results. Most cases of B-ALL/B-LBL are defined by cytogenetic or molecular abnormalities, which form the basis for subclassification, with prognostic and therapeutic implications [3].

The clinical presentation of B-ALL/B-LBL can be rapid or insidious, with symptoms mostly due to bone marrow replacement and including weakness, fever, pallor, petechiae, and bone pain [5-7]. Extramedullary involvement is infrequent (10-15%) and may occur at the onset of the disease or during relapse, leading to varied symptoms and imaging findings depending on the affected organs [6,7].

Most of the available literature on extramedullary involvement in B-ALL/B-LBL comes from case reports and case series, which have described multiple locations, including the head and neck, central nervous system, retroperitoneum, pleura, gastrointestinal tract, genitourinary tract, testicles, breast, skin, and soft tissues [4,8-12]. Mediastinal and orbital involvement has been described in B-ALL/B-LBL, but it occurs more frequently in myeloid leukemias (granulocytic sarcoma, previously known as chloroma) and in T-cell derivatives [8].

According to the literature, extramedullary manifestations are more frequent in relapses, occurring in 27-50% of cases, and may affect one or multiple locations [5]. Some anatomical sites called "sanctuaries" have been described, where leukemic cells have relative protection from chemotherapeutic agents, making them the main sites of relapse in extramedullary involvement, including the testicles, orbits, and central nervous system. On the other hand, the most frequently affected sites at the time of diagnosis are the head and neck, orbit, nervous system, testicles, and skin [6].

Imaging studies in B-ALL/B-LBL usually depend on symptoms and include ultrasound, computed tomography (CT), and magnetic resonance imaging (MRI). Imaging findings interpreted in isolation can be nonspecific, but evaluation in conjunction with laboratory tests and clinical examination can lead to an early diagnostic suspicion.

The presence of masses is usually the main finding in multiple organs [8]. They appear isointense or hypointense on MRI T1-weighted sequences and isointense or hyperintense on T2-weighted sequences, with varying enhancement with contrast medium, and variable restriction on diffusion sequences [7]. Orbital involvement can be accompanied by bone erosion and extension to adjacent structures such as paranasal sinuses and the nasopharynx [8-10]. Testicular involvement can be unilateral or bilateral and is best evaluated by ultrasound, where there will be an increase in size and vascularity on color Doppler, with diffuse or irregular hypoechoic parenchyma [4]. Ovarian involvement is characterized by an increase in size, but its detection is difficult [4]. Soft tissues and skin present masses or nodules, with variable depth and nonspecific characteristics [8]. Table 1 summarizes the extramedullary findings of B-cell lymphoblastic leukemia/lymphoma.

**Table 1:** Summary of findings in extramedullary involvement by B-cell lymphoblastic leukemia/lymphoma

Organs/systems affected by order of frequency	Findings
Skin and soft tissues	Masses, nodules, plaques [4,7].
Central nervous system	Intracranial and spinal epidural masses, meningeal invasion (with pachymeningeal enhancement) [4,7].
Orbit	Masses, proptosis, increased size and enhancement of the optic nerve, hemorrhage, and retinal detachment [10,11].
Testicles and ovaries	Increased size and vascularization [4].
Musculoskeletal	Osteopenia, radiolucent metaphyseal lines, and fractures [4].
Head and neck	Masses of variable location, parotidomegaly, and hypertrophy of the Waldeyer ring [4,7,9].
Abdomen	Masses (renal, vesical, prostatic, in the gastrointestinal tract, peritoneal and retroperitoneal), nephromegaly, involvement of intestinal loops (wall thickening, ulcers, polyps), hepatosplenomegaly, peritoneal thickening, and ascites [4,7,12].
Pleura	Pleural deposits [4,7].
Heart	Pericardial deposits [4].
Breasts	Masses, asymmetries [4].

The findings in our patients were consistent with those reported in the literature, showing a higher frequency of extramedullary involvement in boys under six years of age. All patients had mixed involvement (B-ALL/LBL). The most frequent site of involvement was the soft tissues (especially the frontal region). The second most frequent site of involvement was the central nervous system, with invasion of the meninges and intraspinal masses. There was also the involvement of the orbit, neck, pleura, kidneys, retroperitoneum, presacral space, bladder, and testicles.

## Conclusions

B-cell leukemia is a common malignant neoplasm in childhood, often presenting with nonspecific symptoms and occasionally with extramedullary manifestations. We present a series of cases of patients with extramedullary manifestations of B-cell leukemia and describe their clinical, imaging, and histopathological characteristics. The findings in our patients were consistent with those reported in the literature, showing a higher frequency of extramedullary involvement in boys under six years of age. All patients had mixed involvement with definitive criteria for leukemia in the bone marrow study. The most common sites of extramedullary involvement were soft tissue and the central nervous system. Some clinical characteristics, together with findings on diagnostic imaging, can establish a pattern of presentation that may suggest early diagnosis and avoid treatment delays.
